# Self-Assembled Nanocomposites and Nanostructures for Environmental and Energic Applications

**DOI:** 10.3390/nano13020220

**Published:** 2023-01-04

**Authors:** Tifeng Jiao, Byoung-Suhk Kim, Peizhi Guo, Bingbing Li

**Affiliations:** 1 School of Environmental and Chemical Engineering, Yanshan University, Qinhuangdao 066004, China; 2 Department of Organic Materials & Fiber Engineering, Jeonbuk National University, 567 Baekje-daero, Deokjin-gu, Jeonju-si 54896, Republic of Korea; 3 Institute of Materials for Energy and Environment, College of Materials Science and Engineering, Qingdao University, Qingdao 266071, China; 4 Department of Chemistry and Biochemistry, Central Michigan University, Mount Pleasant, MI 48859, USA

With the rapid development of nanotechnology, nanocomposites and nanostructures have attracted significant attention due to their unique physical and chemical properties and variable functionalities [1,2,3]. Both the various compositions and the construction process of nanostructured composites influence their performances, with application areas ranging from new chemical reactions, organic semiconductors, photovoltaic technology, and photocatalysts to biosensors and energy materials [4,5,6].

This Special Issue aims to provide readers with a compilation of cutting-edge research about self-assembling technology for fabricating nanocomposites and nanostructures.

Self-assembly is a process in which components, either separated or linked, spontaneously form ordered aggregates [7]. Self-assembly can be used to construct various nanostructured materials and composites such as particles, films, gels, and composites [8,9], achieving controllable chemical functionalization and well-defined micro-/nanostructures of nanostructured composites.

Some of these advantages of self-assembled nanocomposites and nanostructures for environmental and energic applications have been developed in this Special Issue.

Yang et al. prepared bright blue fluorescent N-doped graphene quantum dots (N-GQDs) via an ultrasonic-assisted hydrothermal method [10]. The obtained N-GQDs showed excellent photo-bleaching resistance and superior photo-stability at room temperature and in the pH range of 3–8, demonstrating great potential for bioimaging or biomarking applications.

Ordered and disordered mesoporous structures were prepared via self-assembly and low-temperature autoclave methods by Kim et al., and their potential application in a supercapacitor was demonstrated [11].

Yifang Liu and co-colleagues designed a self-supporting, three-dimensional (3D) nanofibrous membrane with a curled pattern, which could increase porosity and reduce pressure drop, and exhibited improved air-filtration performance [12].

A graphene-functionalized complementary terahertz MS, composed of a dipole slot and two graphene-integrated quadrupole slots with different sizes, was proposed to execute selective and active control of dual-band electromagnetic induced reflection (EIR) windows by Gao et al. [13].

Wang et al. adopted the three-dimensional model to simulate the electric field distribution of SiO_2_@Ag composite nanospheres, revealing the SiO_2_@Ag hemisphere’s electric field electrostatic shielding effect [14].

A green tea extract was used to prepare Iron nanoparticles (nFe), and its use in the removal of hexavalent chromium was demonstrated by Jiao et al. [15].

I would like to thank all who have provided original research articles included in this Special Issue. Readers will certainly find other interesting aspects of the implications of self-assembly in the construction of various nanostructured materials and composites by studying the papers presented in this Special Issue of “Self-Assembled Nanocomposites and Nanostructures for Environmental and Energic Applications”.
